# Effect of Simulated Dental Pulpal Pressure Using Fetal Bovine Serum for the Bonding Performance of Contemporary Adhesive to Dentin

**DOI:** 10.3390/polym16091219

**Published:** 2024-04-26

**Authors:** Yitong Li, Masahiko Maeno, Carolina Cecilia Cifuentes-Jimenez, Mei Komoto, Yunqing Liu, Yoichiro Nara, Hidehiko Sano, Pedro Alvarez-Lloret, Monica Yamauti, Atsushi Tomokiyo

**Affiliations:** 1 Department of Restorative Dentistry, Graduate School of Dental Medicine, Hokkaido University, Kita 13, Nishi 7, Sapporo 060-8586, Japan; liyitong@den.hokudai.ac.jp (Y.L.); liuyunqing@den.hokudai.ac.jp (Y.L.); sano@den.hokudai.ac.jp (H.S.); tomokiyo@den.hokudai.ac.jp (A.T.); 2 Department of Adhesive Dentistry, School of Life Dentistry at Tokyo, The Nippon Dental University, 1-9-20, Fujimi, Chiyoda-ku, Tokyo 102-8159, Japankoumoto-ngs@tky.ndu.ac.jp (M.K.); yo-nara@tky.ndu.ac.jp (Y.N.); 3 Department of Stomatology, Faculty of Dentistry, University of Granada, Campus de Cartuja, s/n, 18011 Granada, Spain; carolinaccj@correo.ugr.es; 4 Department of Geology, Faculty of Geology, University of Oviedo, Campus de Llamaquique, s/n, 33005 Oviedo, Spain; pedroalvarez@uniovi.es; 5 Department of Mineralogy and Petrology, Faculty of Sciences, University of Granada, Av. Fuente Nueva, s/n, 18071 Granada, Spain

**Keywords:** microtensile bond strength, simulated pulpal pressure, dentin, adhesives, aging, scanning electron microscopy, Weibull analysis

## Abstract

This study evaluated the effect of simulated pulpal pressure (SPP) conditions and storage time on contemporary adhesive systems’ microtensile bond strength (µTBS) to dentin. Extracted human molars were prepared and randomly divided into four groups according to the adhesives: Clearfil Megabond 2 (CSE), Beautibond Xtreme Universal (BXU), G2-Bond (G2B), and Scotchbond Universal Plus (SBP). Each adhesive group was further divided following the SPP conditions: control with no simulation (SPP-CTR), SPP with distilled water (SPP-DTW), and SPP with fetal bovine serum (SPP-FBS). Resin composite build-ups were prepared, and teeth were stored in water (37 °C) for 24 h (24 h) and 3 months (3 m). Then, teeth were sectioned to obtain resin–dentin bonded beams and tested to determine the µTBS. Data were analyzed using three-way ANOVA, Tukey post hoc tests (=0.05), and Weibull failure analysis. Failure mode was observed using scanning electron microscopy. The µTBS response was affected by adhesive systems, simulated pulpal pressure conditions, and storage time. SPP-CTR groups presented a higher overall bond strength than SPP-DTW and SPP-FBS, which were not significantly different from each other. Only for SBP, the SPP-FBS group showed higher µTBS than the SPP-DTW group. The Weibull analysis showed that the bonding reliability and durability under SPP-DTW and SPP-FBS were inferior to SPP-CTR, and the 24 h bonding quality of adhesives to dentin was superior to that of 3 m. SPP drastically reduced the µTBS of all adhesives to dentin regardless of solution (distilled water or fetal bovine serum). Storage after 3 m also decreased µTBS despite the SPP condition.

## 1. Introduction

The evolution of dental adhesives has achieved significant progress and advances due to simplifying their application steps and technique sensitivity [1]. The self-etch system is the leading type of adhesive in the dental market since it simplifies the technique and shortens the application time compared to the etch-and-rinse adhesive system [2]. In most one-step self-etch (1-SEA) and universal adhesives, all components are incorporated into a single bottle containing hydrophobic and hydrophilic monomers [1]. However, high hydrophilicity is the main detriment of the 1-SEA because it allows fluids from the substrate to diffuse through the dentin [1,2]. The latest and multipurpose version of one-bottle systems, known as the universal multimode adhesive system, can be used in etch-and-rinse, self-etch, and selective-etch strategies with several substrates and is attractive to dentists [1,2].

Dentin is a complex substrate consisting of a mineralized collagen network with tubules allowing pulpal-dentinal fluid flow under a pressure corresponding to 14.1 cmH_2_O [3]. The pulpal-dentinal fluid permeates the space between the odontoblast process and the dentinal tubule wall, referred to as dentinal fluid. It corresponds to an ultrafiltrate of blood plasma composed primarily of water, proteoglycans, tenascin, fibronectin, and the serum proteins albumin, glycoprotein, transferrin, and enzymes [4]. The protein content in the pulpal-dentinal fluid is about one-fifth of the plasma [5]. During the restoration of a vital tooth, the pulpal-dentinal fluid emerges on the dentin surface due to pulpal pressure and immediately dilutes the adhesives’ components [6]. The water content of the pulpal-dentinal fluid can be retained in the adhesive interface, resulting in phase separation, water-tree formation, nanoleakage, poor polymerization, and gap formation, especially at the dentin gingival margin [6,7].

In the short term, the infiltration of fluids may impair the bonding of adhesives to dentin and the durability of restorations. Some studies have found that both pulpal-dentinal fluid and adhesive permeability could hamper the bonding effectiveness between dentin and 1-SEA, resulting in the degradation of the resin–dentin interface due to hydrolysis [6,7]. 1-SEAs are considered semi-permeable membranes that allow subsequent water to contaminate the bonding surfaces under the SPP even after the polymerization [1,2,7]. It is still not well understood how universal adhesives perform in the etch-and-rinse or self-etch mode in varied dentin humidity conditions [8,9,10].

Universal adhesives are 1-SEA that can be utilized following the etch-and-rinse or self-etch mode, thus containing numerous components [1,11]. 2-Hydroxyethylmethacrylate (HEMA) is a hydrophilic monomer essential in dentin bonding procedures. Its presence is vital in adhesive systems to improve their wettability and diffusion through the dentin collagen fibers and prevent phase separation [11]. HEMA is also well known for its protein coagulation effect; previous adhesive studies have shown higher bond strength using human plasma within 24 h [8,9].

In laboratory research on resin–dentin bonding, a dynamic simulated pulpal pressure (SPP) with distilled water (DTW) or saline solution has been widely utilized in an attempt to replicate the oral–dental conditions during the adhesive procedure [6,10,12,13]. However, a more clinically relevant protein-containing solution should be used, which might induce protein-monomer coagulation [8,9,14]. Few researchers utilize human plasma for laboratory experiments because of its problematic operability and feasibility, such as limited storage time [8,15]. Instead, fetal bovine serum (FBS) may be considered a more clinically relevant intramedullary fluid owing to its similar composition to human plasma, especially regarding the proteins [9,14].

Although universal adhesives enhance the simplicity and versatility of adhesive materials, doubts persist regarding their reliability. Still, notable disparities between their laboratory and clinical performance are found [16]. It is imperative to foster comparisons between newly introduced universal adhesives and established standard materials under experimental conditions that imitate relevant clinical variables [12,17]. Therefore, this study aimed to evaluate the influence of simulated pulp pressure (SPP) using distilled water (DTW) or fetal bovine serum (FBS) on the microtensile bond strength to dentin of a two-step self-etch (2-SEA) and three universal adhesives after 24 h and 3 m of water storage. The null hypotheses were (1) different types of adhesive systems, (2) pulpal pressure conditions, and (3) storage time would not affect the bond strength of the tested materials to dentin.

## 2. Materials and Methods

The study regarded an in vitro, prospective, and qualitative–quantitative design. The dependent variable was bond strength to dentin, and the independent variables were adhesive system (4 levels), pulpal pressure condition (3 levels), and storage time (2 levels). The sample size followed the guidance of the Academy of Dental Materials [18].

### 2.1. Teeth Collection and Specimen Preparation

Extracted non-carious human third molars (*n* = 120) were collected after the research project had been approved by the Ethics Committee of the Hokkaido University Faculty of Dentistry (protocol #2018-09) and with the patient’s informed consent. Teeth were cleaned and stored in 0.5 wt.% aqueous chloramine-T solution at 4 °C and used within six months of extraction. The selected teeth were lacking from decay, cracks, restorations, and no enamel or dentin pathology. Each tooth was sectioned perpendicular to the long axis to expose flat mid-coronal dentin using a gypsum model trimmer (Model Trimmer, MT-7; J Morita, Tokyo, Japan) with a water coolant. Subsequently, dentin surfaces were checked for remaining enamel or other defects using a stereoscope. To prepare the samples for SPP, the teeth roots were removed at approximately 2–3 mm below the cementum–enamel junction, creating direct access to the pulp chamber using a low-speed diamond saw (Isomet; Buehler Ltd., Lake Bluff, IL, USA). The pulpal tissue was carefully removed with an endodontic file without touching the dentinal walls. The dentin surfaces were further polished using 600-grit silicon carbide paper (Sankyo-Rikagaku, Okegawa, Japan) under running water for 60 s to produce a standardized smear layer. The teeth in the pulpal pressure group were attached to a Plexiglas (2.0 cm × 2.0 cm × 1.0 cm) platform and sealed with a cyanoacrylate adhesive (Model Repair II Blue, Dentsply-Sankin, Tokyo, Japan). An 18-gauge stainless needle was inserted into the platform connected to a 40 mL syringe filled with DTW or FBS (ThermoFisher Scientific/Gibco, New York, NY, USA) to simulate the 15 cmH_2_O pulpal pressure [13,14].

### 2.2. Bonding Procedures

The adhesive systems tested in this study are listed in Table 1. The teeth were randomly separated into four main groups following the adhesive systems. Each adhesive group was randomly divided into three subgroups based on the SPP conditions: Absence of SPP (control, SPP-CTR), SPP with DTW (SPP-DTW), and SPP with FBS (SPP-FBS). The FBS solution was diluted in physiological saline with a 1:3 ratio [9,14]. Each adhesive was applied based on the manufacturers’ instructions. Resin composite (Clearfil^TM^ AP-X, Kuraray Noritake Dental, Okayama, Japan) blocks (4 mm) were incrementally built up, and each 1 mm resin increment was light-irradiated using an LED light-curing unit at ≥1200 mW/cm² (G-Light Prima-II plus, GC Corporation, Tokyo, Japan). The prepared teeth were stored in DTW at 37 °C for 24 h and 3 m.

### 2.3. Microtensile Bond Strength (μTBS) Test

After water storage, each bonded tooth was vertically sectioned into a cross-sectional area of approximately 1 mm^2^ to obtain resin–dentin specimens employing the non-trimming technique with a low-speed diamond saw (Isomet 1000, Buehler, Lake Bluff, IL, USA) under running water. Nine central resin–dentin beams per tooth were selected from the central part for the μTBS test. The beams were fixed to Ciucchi’s jig with a Model Repair II Blue adhesive (Dentsply-Sankin, Tokyo, Japan). They were subjected to tensile force employing a 500-N load cell at a 1 mm/min crosshead speed in a desktop testing apparatus (EZ-S, Shimadzu Co., Kyoto, Japan) until fracture occurred. Each beam was tested within 5 min after removal from water storage to prevent the sample from drying [18]. The bond strength was calculated and expressed in MPa, and the mean value of 9 central beams derived from each tooth represented the μTBS of that tooth, generating 5 data points for each group (*n* = 5).

### 2.4. Fracture Mode Analysis

The fractured surfaces of the resin composite and dentin sides were room-dried overnight. They were then attached to aluminum stubs and coated with Pt-Pd alloy (E-1030, HITACHI, Tokyo, Japan) for 120 s and observed using a scanning electron microscope (SEM, S-4000, HITACHI) at an accelerating voltage of 10 kV. Failure modes were classified into adhesive failure, cohesive failure within resin composite, cohesive failure within dentin, or mixed failure [18].

### 2.5. Observation of the Resin-Dentin Interface

One tooth per group was bonded according to the μTBS test preparation. After 24 h water storage, the teeth were cut into slabs parallel to the long axis. Two slabs from the central part were selected and prepared for detection. All slabs were subsequently polished with a series of ascending SiC waterproof papers (600-, 800- and 1000-grit, Sankyo-Rikagaku) under copious water and descending grit diamond pastes (6, 3, and 1 μm, DP-Paste; Struers, Ballerup, Denmark). All the slabs were ultrasonicated in DTW for 5 min and treated with 1 M hydrochloric acid (HCl) for 10 s, followed by a 5.25 wt.% sodium hypochlorite (NaOCl) solution for 5 min. The specimens were dehydrated in an incubator for 24 h, sputter-coated with Pt-Pd for 120 s, and observed with SEM.

### 2.6. Degree of Conversion Analysis

A modular confocal Raman spectrograph was used to investigate the adhesives’ degree of conversion (DC) under cured and uncured conditions. Spectra were obtained using a JASCO NRS-5100 spectrometer (Jasco Inc., Easton, MD, USA). Two drops of each adhesive were poured into a circular Teflon sample holder (10.0 mm diameter × 4.0 mm depth) and placed under the microscope on a computer-controlled XYZ stage to obtain the uncured monomer spectra. A near-infrared diode laser (785 nm) kept at 500 mW was employed to induce the Raman scattering. Spectra were acquired between 1000 and 1800 cm^−1^ using an exposure time of 5 s and 10 accumulations with an average spectral resolution of 1.6 cm^−1^. Each adhesive sample was polymerized with an LED light-curing unit (G-Light Prima-II plus, GC Dental Corporation, Tokyo, Japan) following the manufacturer’s instructions, and the cured measurements were taken. Three specimens were employed for spectral analyses for each material. The DC values were calculated by determining the polymerized specimens regarding the changing peak amplitude ratio of the absorbance aliphatic C=C at 1638 cm^−1^ and the internal reference peak of aromatic C=C at 1608 cm^−1^ [19,20]. The intensity/amplitude of the reference peaks was determined following the methodology described elsewhere [21]. A region of the spectra between 1590 and 1660 cm^−1^ was selected, and the baseline was corrected. After spectrometric analyses, the DC was calculated as follows:

$$\mathrm{DC}\;(\%){\;}_{\mathrm{amplitude}}=[1-\frac{{Cured\;C=C1638\;cm}^{-1}/{Cured\;C=C1608\;cm}^{-1}}{{Uncured\;C=C1638\;cm}^{-1}/{Uncured\;C=C1608\;cm}^{-1}}]\times 100$$

### 2.7. Statistical Analysis

Bond strength data were checked for normality (*p* = 0.231) using Shapiro–Wilk and homogeneity of variance (*p* = 0.130) using Brown–Forsythe. The effect of the three independent variables on the μTBS of the adhesives to dentin values was analyzed using three-way ANOVA and Tukey HSD post hoc tests. All statistical analysis was performed using SPSS 27.0 for Windows (IBM SPSS, Chicago, IL, USA) with a level of significance set at α = 0.05. Additionally, to estimate the bonding reliability qualitatively based on μTBS, Weibull parameters—Weibull modulus (Wm) and Weibull stress at a probability of failure of 10% (PF10) and 90% (PF90)—were determined by using spreadsheet software (Excel 2016 for Windows; Microsoft, Redmond, WA, USA) at the set 5% level of significance [22]. When the specimens failed to obtain the beam specimen during preparation (pretesting failure—ptf), the specimen’s value was replaced with a random value between zero and the lowest data point measured for the respective group for the Weibull analysis [23]. The degree of conversion of the adhesives followed a normal distribution (*p* = 0.220) and equal variance (*p* = 0.780) was analyzed with a one-way ANOVA test with a level of significance set at α = 0.05.

## 3. Results

### 3.1. μTBS Test

No pretest failures were registered, and the μTBS results are shown in Table 2. Bond strength results were significantly affected by adhesive systems (*p* < 0.001), storage time (*p* < 0.001), and simulated pulpal pressure condition (*p* < 0.001) but not by the interaction between the three factors (*p* = 0.379). There were statistically significant two-factor interactions between the independent variables (*p* < 0.002). The effect of adhesives was dependent upon the level of simulated pulp pressure condition (*p* < 0.001) and storage time (*p* = 0.002). The SPP-CTR groups of the adhesive systems demonstrated superior μTBS values to those under SPP, regardless of the solution (DTW or FBS). Storage time significantly decreased μTBS of the adhesives despite the solution utilized (*p* < 0.05). Interestingly, the μTBS of SBP increased when stored under SPP-FBS at 24 h and 3 m compared to the SPP-CTR condition (*p* = 0.005).

### 3.2. Failure Mode Observation

**Figure 1:**
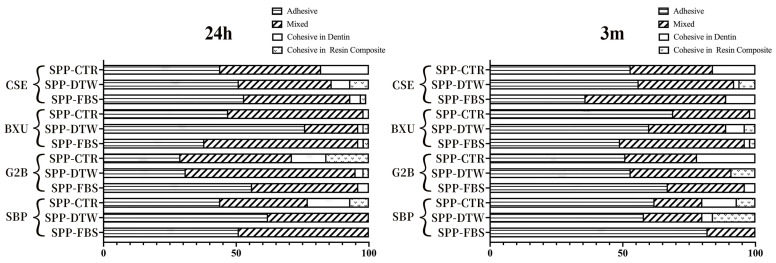
Bar graphs represent the percentage distribution of failure mode within each group following different storage periods.

**Figure 2:**
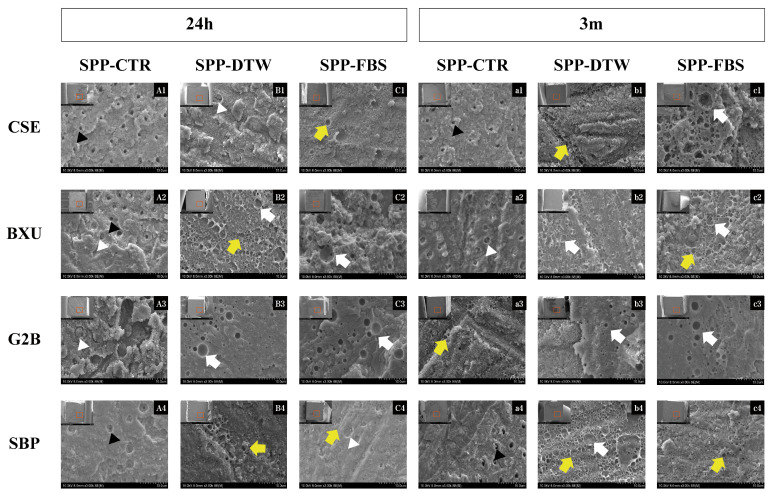
Representative SEM images of the dentin part of each group’s adhesive failure after 24 h and 3 m at ×3000 magnification. On the top left corner of each image (**A1**–**c4**), there is a small image correspondent to the fractured surface of the dentin beam after the bond strength test. The red squares indicate the sites where the high-magnification images were obtained. Images **A1**–**C4** exhibit the fractured surfaces after 24 h storage for each adhesive (row) and SPP condition (column). Images **a1**–**c4** display the fractured surfaces after 3 m storage for each adhesive (row) and SPP condition (column). The white arrows indicate notable and large bubbles in the adhesive layer. The yellow arrows indicate numerous small voids in the adhesive layer. The black arrowheads indicate the dentinal tubules’ openings and the white arrowheads depict partially occluded dentinal tubules.

The percentage distribution of failure modes detected in this study is shown in Figure 1. No premature failures occurred during the specimen preparation, and the failure mode varied per different adhesive systems and simulated pulpal pressure conditions. After 24 h storage, the predominant failure mode was adhesive except for BXU under SPP-FBS (58% mixed failure) and G2B under SPP-CTR (42% mixed failure) and SPP-DTW (64% mixed failure) conditions. There was an increase in adhesive failure ranges after 3 m storage, except for the CSE under SPP-FBS, BXU under SPP-DTW, and SBP under SPP-DTW groups, which presented a slight decrease (respectively, 36%, 60%, and 58%) in adhesive failure. When representative fractured specimens were examined (Figure 2), tubule apertures could be detected in all control groups’ adhesive failure within the adhesive layer. On the contrary, numerous voids appeared in the adhesives’ layers under the SPP conditions, especially for the SPP-DTW groups. Resin tag plugs were found in the adhesive layer (Figure 2A2,a2,B1,A3,C4)

### 3.3. Interface Observation

**Figure 3:**
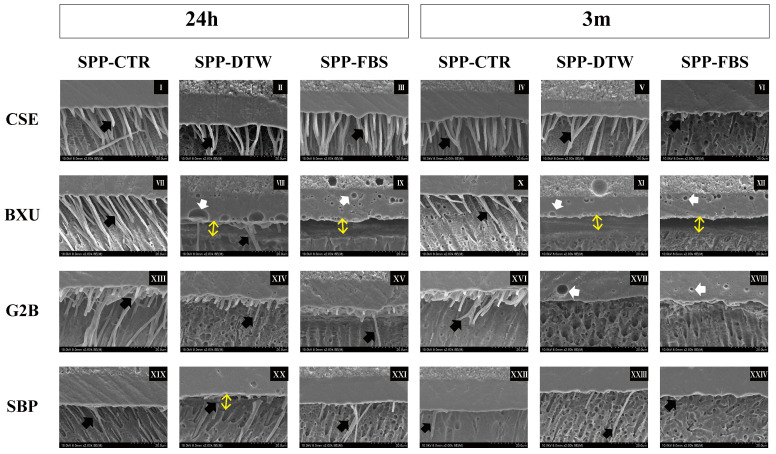
Representative SEM images of adhesives’ interfaces (2000×). Black arrows indicate resin tags. Double-ended yellow arrows represent gap formation at the resin–dentin interface. White arrows depict bubbles in the adhesives’ layer. The adhesive interfaces after 24 h storage are shown on images **I**–**III** (CSE), **VII**–**IX** (BXU), **XIII**–**XV** (G2B), and **XIX**–**XXI** (SBP). The 3 m storage images are represented in images **IV**–**VI** (CSE), **X**–**XII** (BXU), **XVI**–**XVIII** (G2B), and **XXIII**–**XXIV** (SBP). The ascending order of numbers refers respectively to SPP-CTR, SPP-DTW, and SPP-FBS for each adhesive at 24 h and 3 m.

SEM images of the resin–dentin interfaces are presented in Figure 3. For CSE groups, resin tags were long and densely detected under all conditions except FBS-3 m, where only short resin tags were observed. Resin tags were only observed in BXU control groups, and several gaps and bubbles were found in the adhesive layer under other SPP conditions. Resin tags in G2 B groups either lacked or appeared as very short projections and were scarcely distributed along the observed area, except in the CTR-24 h groups, which presented long resin tags. Fewer round voids appeared within the layer of G2B under SPP-DTW and SPP-FBS conditions in 3 m. In both SPP conditions, resin tags were hardly detected in SBP adhesive interfaces, and gaps were observed between the adhesive layer and dentin.

### 3.4. Bonding Reliability and Durability

**Figure 4:**
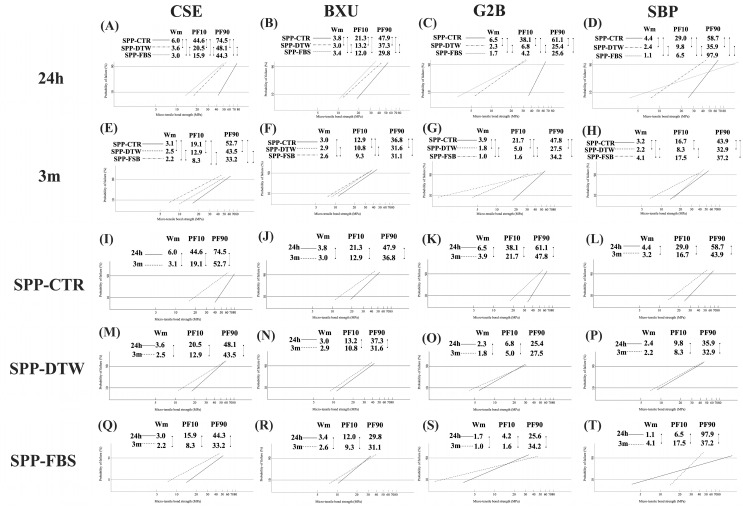
Differences in Weibull parameters among 24 h (**A**–**D**) and 3 m (**E**–**H**) storage time separately for each adhesive system; *n* = 45. Differences in Weibull parameters between storage time among all adhesives under SPP-CTR (**I**–**L**), SPP-DTW (**M**–**P**), and SPP-FBS (**Q**–**T**); *n* = 45. Two groups connected with a straight line indicate a significance at *p* < 0.05. Wm, Weibull modules; PF10 and PF90, the Weibull stress value in MPa for 10 and 90% probability of failure level.

Figure 4 shows the differences in the Weibull parameters among four adhesives under three SPP conditions and two storage times. The listed parameters are the Wm, PF10, PF90, and the number of specimens [22]. In the 24 h data, there were significant differences among all SPP conditions in Wm values with the decreased sequence of SPP-CTR > SPP-DTW > SPP-FBS, except for BXU (SPP-CTR > SPP-FBS > SPP-DTW). Concerning the 24 h data for PF10, all adhesives showed significant differences among the SPP conditions following the decreasing order of SPP-CTR > SPP-DTW > SPP-FBS; however, no significant differences were found for the PF10 values between SPP-DTW and SPP-FBS. Overall, SPP conditions decreased the bonding reliability and durability among all adhesives tested in this study.

On the other hand, 3 m data revealed that significant differences in Wm values were found among all SPP conditions except for BXU, which did not show SPP-CTR and SPP-DTW. Moreover, the Wm values similarly decreased in G2B and CSE as SPP-CTR > SPP-DTW > SPP-FBS. The PF10 values of BXU, G2B, and CSE significantly decreased as SPP-CTR > SPP-DTW > SPP-FBS. In the case of SBP, the maximum Wm and PF10 values were found for the SPP-FBS condition, while the minimum values were detected in the SPP-DTW condition.

Mainly, 3 m of storage time resulted in lower Wm and PF10 than 24 h storage, except for Wm values of BXU and SBP under SPP-DTW. Intriguingly, FBS positively affected the bonding reliability and durability of SBP after 3 m storage compared to DTW.

### 3.5. DC

Table 3 shows the DC (%) obtained from each adhesive’s intensity/amplitude of the reference peaks. G2B presented the lowest DC values (90.13%) among the adhesives (range of adhesives’ DC: 93.6 to 96%). There was no statistically significant difference between the DC values of BXU, CSE, and SBP (*p* > 0.05).

## 4. Discussion

This study evaluated the effect of different adhesive materials under simulated pulpal pressure conditions and storage time on the μTBS to dentin. As statistically significant differences were detected in the bond strength of the different types of adhesive systems (a 2-SEA and three universal adhesives) to dentin, the first null hypothesis (1) was rejected. The SPP conditions, regardless of the solutions—DTW or FBS—negatively decreased the bond strength of the adhesives to dentin; thus, the second null hypothesis (2) was rejected. All adhesive materials showed lower bond strength after 3 m of storage than 24 h, therefore rejecting the third null hypothesis (3).

The chemistry of resin-based adhesives is crucial for the longitudinal bonding stability with dental hard tissues, especially in contemporary universal adhesives, a blend of acidic functional monomers, hydrophilic and hydrophobic monomers, solvents, fillers, activators, initiators [11,24]. CSE presented the leading μTBS of all adhesives in each experimental condition. This performance is attributed to its extra hydrophobic resin layer and its functional monomer, 10-MDP, in its primer composition [2,7]. 10-MDP interacts with dentin by self-assembling with calcium and forming Ca-MDP, a stable compound salt with a decreased dissolution rate that can stabilize dentin collagen fibers [1,2,11]. The 10-MDP has consistently been recognized as the most favorable monomer for chemically bonding to hydroxyapatite in enamel or dentin [25]. It is unlikely that, in some universal adhesives (e.g., BXU and G2B), reliable long-term bonding is not guaranteed due to several factors, such as complex chemical composition, bonding strategy, and dependence on the dental substrate [26,27]. Dental adhesives also contain hydrophobic dimethacrylate monomers like UDMA and TEGDMA, which form highly cross-linked polymers that enhance the mechanical strength of the cured materials [27,28]. However, the resulting polar-ether linkages and hydroxyl groups allow water absorption [28]. The TEGDMA content in BUX may have accounted for 24 h and 3 m low bond strength. Even though the bonding agent of G2B contains a hydrophobic monomer (UDMA), G2B primer also contains 4-MET and 10-MDTP solvated by acetone that evaporates quickly, leaving too much water on the dentin surface [24], probably leading to low bond strength under the SPP conditions. 1-SEA and universal adhesives’ intricate compositions make HEMA’s addition and optimal concentration problematic [24]. HEMA, a hydrophilic monomer easily mixed with ethanol, is commonly utilized as a co-solvent in dental adhesive formulations to improve monomer miscibility [11,24,28]. CSE performance confirms that HEMA-containing adhesives can attain bonding effectiveness to the dentin, as previously stated [1,2]. HEMA’s capacity to increase other monomers’ conversion and mechanical properties and promote long resin tag formation may explain CSE’s superior bonding performance [24,28].

The ability of self-etch adhesives to remove or dissolve the smear layer more promptly correlates with their pH [1,2]. G2B (pH = 1.5) and CSE (pH = 2.0) are classified as intermediately strong, BXU (pH = 2.4) as mild, and SBP (pH = 2.7) as ultra-mild self-etch adhesives [1,2], but they did not perfectly align with their expected etching capability (Figure 3). Notably, SBP exhibited the anticipated when SPP-FBS was utilized (Figure 3XXIV) but not in the experimental condition of 24 h of storage, in which numerous and lengthy resin tags were observed (Figure 3XIX). Although low-pH adhesives promote effective smear layer dissolution, this procedure also accelerates water diffusion from dentinal tubules to dentin surfaces, risking excessive fluid contamination on the adhesive surface during the bonding procedure [1,7,26]. In G2B adhesive, the fluids (DTW and FBS) used to simulate pulpal pressure most probably permeated the dentinal tubules and contaminated dentin surfaces, thus forming numerous voids observed in SEM images (Figure 2B3,C3,a3,b3,c3 and Figure 3XVII,XVIII) and compromising the physical properties of the adhesive interface. In addition, this adhesive also underwent polymerization, denoting the lowest DC (Table 3).

Dentin moisture, exacerbated by water diffusion from 1-SEA and universal adhesives, poses a contradictory challenge regarding the HEMA content in these adhesives [2,28]. The main drawback of using HEMA is its water attraction from the substrate and oral environment. This HEMA characteristic impedes humidity control and water evaporation from the adhesive layer, reducing monomer dilution and polymerization degree [2,11,28]. HEMA-free adhesives (i.e., BXU and G2B) have been formulated to overcome HEMA’s shortcomings. However, the negative interaction between hydrophobic and hydrophilic monomers leads to phase separation in HEMA’s absence, adversely influencing adhesives bonding to dentin [2,7]. Phase separation is a complex phenomenon commonly occurring in HEMA-free 1-SEA adhesives [7,11]. As the solvents (ethanol or acetone) evaporate, the water separates from the other adhesives’ components, and several droplets form in the adhesive layer. The water droplets remain entrapped in the polymerized adhesive layer [7]. The droplets contribute to bond degradation as much as the permanence of water inside the droplets may also affect the bonding [2,7]. This phenomenon most likely explains the poor mechanical behavior of BXU and G2B, especially under SPP, when the phase separation might have taken place, compromising dentin bonding. The poor bonding behavior of BXU and G2B over time is corroborated in the literature [19,20]. Moreover, HEMA-free acetone-based adhesives make water contamination during bonding even more critical. Acetone volatilizes much faster than water, making dentin water removal complex, raising the water droplets’ entrapment into the polymerized adhesive layer [2,7]. Consequently, it is recommended that those adhesive types be strongly air-dried [2].

The fluids (DTW or FBS) utilized in the SPP condition during the bonding procedure noticeably influenced the bond strength of adhesives to dentin after 24 h and 3 m storage. Although DTW is commonly used in SPP investigations [13,19], chemical variations in adhesive compositions and their interaction with pulpal-dentinal fluid prevent DTW from reflecting the interaction between adhesive and this fluid. Unlike water, FBS contains growth factors, hormones, carbohydrates, and proteins, thus resembling dentinal fluid [9,14]. In this study, SPP-FBS and SPP-DTW conditions had similar effects on adhesives’ bond strengths at both storage periods, except for CSE (24 h) and SBU (24 h and 3 m). Regarding CSE (3 m), FBS provoked a significant decrease in bond strength. Contrarily, FBS benefited the bond strength of SBP over water, which is corroborated by a previous report [9]. SBP has been suggested to coagulate protein within the dentinal tubules [9]. Denser than water, FBS may take longer to diffuse to the dentin surface, reducing water influx from dentinal tubules to the adhesive interface during bonding [9,14].

According to the International Organization for Standardization (ISO) 11405:2003 guidelines [29], Weibull analysis is a proper qualitative evaluation method for measuring bonding reliability. Concerning bond strength characterization, two principal parameters are utilized by this analysis: the Wm, which can predict the bonding reliability, and the Weibull stress values, which can evaluate the performance of a bond at some invariable percentage level (10% (PF10), 63.2%, and 90% (PF90) levels), causing a failure [22,29]. It has been stated that high Wm and Weibull stress values indicate considerable bonding reliability and durability [22,30]. A high Wm is preferable for materials since it indicates superior homogeneity in the flaw population and a more reliable failure mode prediction [22,30]. The PF10 might be a more relevant parameter than a mean value and PF90, as low PF10 values may reflect early failures under low load in the clinical situation, being more critical than high values (PF90), which relates to a few cases of catastrophic rare situations [31]. Based on the current results, the Wm at 24 h (Figure 4A–D) and 3 m (Figure 4E,G) indicated that the bonding reliability under SPP-DTW and SPP-FBS conditions was inferior to SPP-CTR, except for BXU and SBP after 3 m storage. Interestingly, SBP produced an increased Wm after 3 m storage under SPP-FBS condition, demonstrating improved bonding reliability (Figure 4H). Among all adhesives, the PF10 values indicated that the bonding durability in SPP-FBS was lower compared to the other SPP conditions, except for SBP under SPP-FBS after 3 m storage. Additionally, bonding reliability and durability at 24 h were significantly higher than at 3 m.

Upon polymerization, the adhesives create resin tags in dentinal tubules and through inter- and intratubular hybridization, thus micromechanically bonding to dentin and forming the hybrid layer [2]. Hence, a high DC is desirable for durable bonding [24]. Previous findings report an association between DC and bond strength [32,33]. However, this association does not imply causation, as other variables influence the bond strength of adhesives to dentin [32,33,34]. The results of this current study agree with Tichy et al. [33], who reported a particular association between the DC and long-term bond strength. In the present study, an association between the low DC of G2B and its poor bonding durability under both SPP conditions could be observed. Also, the high DC of SBP could be related to its improved bonding under the SPP-FBS condition. The DC of monomers depends on the chemical composition and polymerization conditions [27]. The primer of G2B contains numerous acidic monomers (Table 1), which could interfere with monomer conversion [27]. Additionally, the presence of 4-MET is related to a lesser capability to create stable monomer-Ca salts [25]. SBP and Scotchbond Universal, its predecessor, have been extensively studied, and their DC has been reported high [27,32,34,35,36]. Scotchbond Universal’s high DC has been ascribed to its relatively low solvent content [36] and application mode, which requires a gently air-blowing until the adhesive film no longer moves on the dentin surface to promote sufficient solvent evaporation and guarantee an appropriate polymerization [2].

While laboratory testing of the durability of bonds does not perfectly translate to clinical settings and outcomes [16], bond strength remains the primary property of screening newly introduced adhesive materials and addresses clinically relevant variables [17]. Considering the results of the present study, as SPP conditions influenced the bond strength of adhesives, particularly the universal type, clinicians should carefully acknowledge the presence of pulpal pressure and pulpal-dentinal fluid when restoring a cavity with adhesive materials. Some strategies have been proposed to overcome the pulpal pressure and pulpal-dentinal fluid issues, such as the active application of universal adhesives to allow solvent evaporation [7], an effective polymerization of the adhesive [1,2], and the application of an extra hydrophobic resin layer on the thin adhesive layer are featured as clinically feasible procedures [1,2,26]. An extra hydrophobic resin layer can overcome the high hydrophilicity of universal adhesives, reducing the pulpal-dentinal fluid’s water uptake and ultimately improving those adhesives’ performance [37,38,39]. Nevertheless, adding an extra hydrophobic layer did not improve the 5-year clinical performance of Scotchbond Universal used to restore non-carious cervical lesions [40]. Well-designed clinical trials need to be conducted to address the clinical effectiveness of this bonding strategy with other universal adhesive systems.

Due to limitations, this study’s conclusions should be considered cautiously. Only resin–dentin medium-term bond strength and DC were investigated. More extended storage periods and additional physicochemical properties (e.g., collagen cross-linking, enzymatic activity, measurement of leachable components, water sorption/solubility, in situ degree of conversion) must be addressed to unravel the relation between pulp–dentinal fluid during the adhesive procedure and their effect on bonding durability. The chemical reactions between dentin collagen, dentinal fluid proteins, and adhesive components should be studied, particularly those of Scotchbond Universal Plus. Although in vitro SPP is laborious, it is a highly relevant experimental condition. It should be included in screening tests aiming at evaluating adhesive materials. No experimental condition can accurately reproduce the oral environment concerning food intake, precise mastication pattern, saliva content, flow, temperature, and pH fluctuations. Thus, clinical trials are mandatory to validate adhesive interfaces’ long-term durability and reliability.

## 5. Conclusions

Considering the experimental conditions of this study, the following conclusions could be drawn:

– The studied factors, (1) different adhesives, (2) pulpal pressure conditions, and (3) storage time, affected the bond strength of adhesives to dentin, therefore rejecting the related null hypotheses.
– Different adhesive systems produced distinct bond strength to dentin under the proposed experimental conditions.
– Clearfil SE Bond 2 achieved the highest bond strength at 24 h of storage when no pulpal pressure was simulated. G2-Bond produced the lowest bond strength after 3 months of storage when pulp pressure was simulated, regardless of the solution used.
– Overall, the simulated pulp pressure and the 3 months of storage decreased the bond strength of the adhesive systems to dentin.
– The effect of simulating pulpal pressure with different fluids depended on the adhesive. Interestingly, the fetal bovine serum increased the bond strength reliability of Scotchbond Universal Plus after 3 months of storage.

## 6. Clinical Significance

The presence of fluids on dentin surfaces impairs the bonding procedure and can jeopardize the durability and reliability of resin–dentin bonds. To minimize restoration failures, dentists should recognize the pulp–dentinal fluid flow through dentinal tubules on dentin surfaces. The clinicians should also acknowledge how this fluid interacts with adhesive systems, follow the manufacturer’s adhesive application instructions, and periodically monitor their patients’ restorations.

## Figures and Tables

**Table 1:** Adhesive systems, manufacturers, pH, composition, and application procedures.

Adhesives; Manufacturers; Abbreviations	pH	Composition *	Application Procedures
Beautibond Xtreme Universal; Shofu INC., Kyoto, Japan; BXU	2.4	Acetone, water, Bis-GMA, carboxylic acid monomer, TEGDMA, organophosphate monomer, acid-resistant silane coupling agent.	1. Apply the adhesive and leave for 10 s. 2. Gently air-blowing for 3 s and blow strongly for 7 s. 3. Light-cure for 10 s.
Clearfil Megabond 2; Kuraray Noritake Dental Co., Tokyo, Japan; CSE	2.0	Primer:10-MDP.HEMA, hydrophilic aliphatic, dimethacrylate, dl-CQ, water. Bond: 10-MDP, Bis-GMA, HEMA, dI-CQ, hydrophobic aliphatic dimethacrylate.	1. Apply the primer and leave for 20 s. 2. Gentle air-blowing > 5 s. 3. Apply the bond. 4. Gentle air-blowing to make the film uniform. 5. Light-cure for 10 s.
G2-Bond Universal; GC Dental Corp., Tokyo, Japan; G2B	1.5	Primer: 4-MET, 10-MDP, 10-MDTP, dimethacrylate monomer, acetone, water, initiators, fillers. Bond: dimethacrylate monomer, Bis-GMA, filler, photoinitiator.	1. Apply the primer and leave for 10 s. 2. Dry with moderate air-blow for 5 s. 3. Apply the bond. 4. Gentle air-blowing to make the film uniform. 5. Light-cure for 5 s.
Scotchbond Universal Plus; 3M Oral Care, Seefeld, Germany; SBP	2.7	10-MDP, Vitrebond TM, Co-polymer, HEMA, dimethacrylate resins, filler, initiators, ethanol, water.	1. Apply the adhesives and leave for 20 s. 2. Gently air-blowing > 5 s until it does not move. 3. Light-cure for 10 s.

* Information as provided by the manufacturers. Abbreviations: Bis-GMA: bisphenol-A-glycidyl methacrylate; TEGDMA: triethylene glycol dimethacrylate; 10-MDP: 10-methacryloyloxydecyl dihydrogen phosphate; HEMA: 2-hydroxyethylmethacrylate; dl-CQ: dl-camphorquinone; 4-MET: 4-methacryloxyethyl trimellitic acid; 10-MDTP: 10-methacryloyloxydecyl dihydrogen thiophosphate.

**Table 2:** Mean (S.D.) of the bond strength (MPa) of the adhesives to dentin following different experimental conditions.

	Simulated Pulpal Pressure		
	Control	Distilled Water	Fetal Bovine Serum
**24** **h**			
BXU	34.80 (4.45) D	25.14 (6.77) E,F	20.94 (4.51) F,G
CSE	60.25 (6.57) A	34.35 (3.63) D	29.87 (3.08) D,E
G2B	50.18 (3.97) B	15.80 (4.57) G,H	13.83 (5.62) H
SBP	44.20 (6.27) C	22.44 (4.94) F,G	35.21 (8.70) D
**3** **m**			
BXU	24.75 (3.00) c,d	21.01 (2.75) d,e	19.94 (2.49) e
CSE	35.71 (4.04) a	27.83 (3.30) b,c	20.15 (1.34) e
G2B	33.02 (7.64) a	15.33 (3.93) f	11.71 (5.90) f
SBP	30.28 (2.23) b	20.07 (1.53) e	27.49 (2.84) b,c

Different capital letters indicate statistically significant differences between groups after 24 h of storage (*p* < 0.05). Different small letters depict statistically significant differences between groups after 3 m of storage (*p* < 0.05). The mid-term storage (3 m) significantly decreased the bond strength of all materials (*p* < 0.003).

**Table 3:** Mean (S.D.) of the degree of conversion (%) of the tested adhesives according to the peak intensity.

Adhesive Systems	Intensity/Amplitude
BXU	93.665 (3.313) A,B
CSE	95.689 (1.588) A,B
G2B	90.127 (1.436) B
SBP	96.007 (1.284) A

Different letters indicate statistically significant differences between adhesives (*p* < 0.05).

## Data Availability

Data are contained within the article.
